# Does Clear Corneal Cataract Surgery Influence Conjunctivochalasis?

**DOI:** 10.18502/jovr.v15i2.6749

**Published:** 2020-04-06

**Authors:** Tatsuya Mimura, Michiko Iida, Hidetaka Noma, Yuko Kamei, Aki Kondo, Maiko Yoshida, Manami Oguri, Yuka Tanaka, Atsushi Mizota

**Affiliations:** ^1^ Department of Ophthalmology, Teikyo University School of Medicine, Tokyo, Japan; ^2^ Department of Ophthalmology, Tokyo Women's Medical University Medical Center East, Tokyo, Japan; ^3^ Department of Ophthalmology, University of Tokyo Graduate School of Medicine, Tokyo, Japan; ^4^ Department of Ophthalmology, Hachioji Medical Center, Tokyo Medical University, Tokyo, Japan

Dear Editor,

Conjunctivochalasis is a common ocular condition characterized by excess conjunctival folds and is associated with aging.^[1,2,3,4]^ We previously demonstrated that the progression of conjunctivochalasis after sclerocorneal tunnel incisions was associated with the axial length of the eyeball and placement of conjunctival sutures.^[4]^ Conjunctival chemosis occurs more frequently in cases of sclerocorneal tunnel incisions than in cases of corneal incisions.^[5]^ This suggests that a clear corneal incision may induce less postoperative conjunctival inflammation compared to a transconjunctival sclerocorneal incision. Therefore, we evaluated the influence of clear corneal incision in cataract surgery on the severity of conjunctivochalasis based on a previously reported grading scale.^[1]^

This study was conducted in accordance with the tenets of the Declaration of Helsinki and was approved by the Institutional Review Board. The inclusion criteria were: age >40 years; nuclear cataract grades II–IV based on the Emery–Little classification; and uncomplicated phacoemulsification surgery. A total of 83 patients (83 eyes) who underwent mini-incision (2.4 mm) phacoemulsification for corneal wound were enrolled, including 43 men and 40 women aged 72.3 ± 9.8 years (mean ± standard deviation), with an age range of 42–88 years.

The severity of conjunctivochalasis was assessed using a modified grading system as previously described.^[1,2]^ Cataract surgery was performed via a clear corneal incision created with a 2.4 mm knife at the superior or temporal position. We evaluated the severity of conjunctivochalasis after one week and one, three, and six months postoperatively. The total conjunctivochalasis score significantly increased after postoperative month 3 (*P* = 0.0017) but decreased after postoperative month 6 (*P* = 0.5806) [Figure 1]. Progression of conjunctivochalasis was defined as ≥ 2-point increase in the total conjunctivochalasis score. Table 1 shows the results of the multivariate analysis using a stepwise selection of factors associated with progression of conjunctivochalasis. The baseline total conjunctivochalasis score was significantly associated with the progression of conjunctivochalasis (odds ratio = 0.90, *P* = 0.0001).

In this study, the postoperative total conjunctivochalasis score increased after postoperative week 1 but subsequently returned to the preoperative level. Additionally, the preoperative severity of conjunctivochalasis was an independent determinant of the postoperative progression of conjunctivochalasis. These results suggest that a clear corneal incision did not change the severity of conjunctivochalasis.

The severity of conjunctivochalasis showed a significant increase after the first postoperative week, which was probably due to postoperative conjunctival inflammation, as there was a subsequent return to baseline. We found that the axial length influences the severity of conjunctivochalasis.^[3]^ Our previous study had also demonstrated that the axial length was independently associated with the grade of conjunctivochalasis after adjustment for age.^[3]^

In conclusion, our results showed no significant change in the severity of conjunctivochalasis after 24 postoperative weeks despite a transient early increase. This article complements our previous study on the progression of conjunctivochalasis after sclerocorneal tunnel incisions.

**Figure 1:**
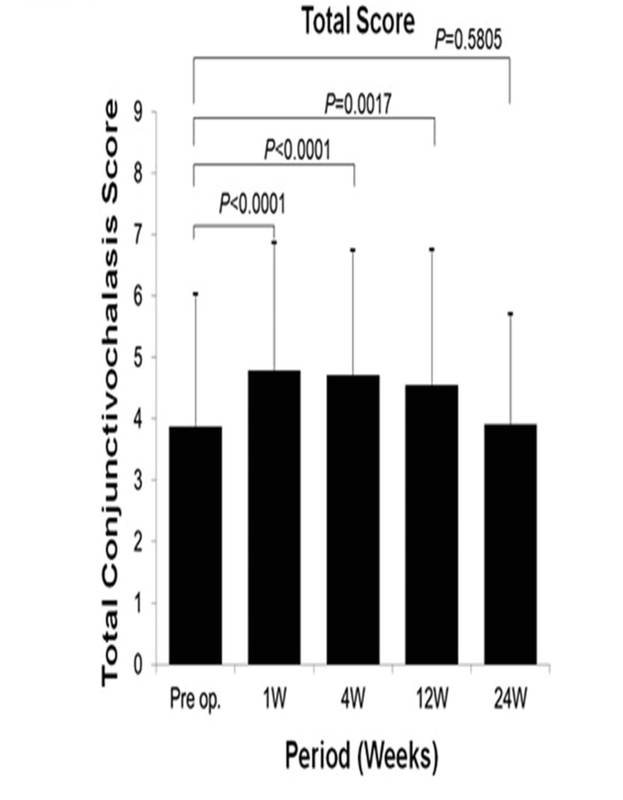
Mean postoperative total conjunctivochalasis score at each time point.

##  Financial Support and Sponsorship

The Ministry of Education, Culture, Sports, Science and Technology of Japan provided financial support in the form of a Grant-in-Aid for Scientific Research (16K11332). The sponsor had no role in the design or conduct of this research.

##  Conflicts of Interest

There are no conflicts of interest.
